# Mechanism of Curcumol Targeting the OTUB1/TGFBI Ubiquitination Pathway in the Inhibition of Angiogenesis in Colon Cancer

**DOI:** 10.3390/ijms26104899

**Published:** 2025-05-21

**Authors:** Yimiao Zhu, Wenya Wu, Dahai Hou, Yu Zhao, Jinshu Ye, Lizong Shen, Tong Zhao, Xiaoyu Wu

**Affiliations:** 1 First Clinical Medical College, Nanjing University of Chinese Medicine, Nanjing 210046, China; zhuyimiao@njucm.edu.cn (Y.Z.);; 2 School of Integrated Medicine, Nanjing University of Chinese Medicine, Nanjing 210023, China; 3 Nanjing Medical University, Nanjing 211166, China

**Keywords:** curcumol, OTUB1, TGFBI, angiogenesis, colon cancer

## Abstract

Tumor angiogenesis and metastasis are critical processes in the progression of colon carcinoma. Curcumol, a bioactive sesquiterpenoid derived from curcuma, exhibits anti-angiogenic properties, though its underlying mechanisms remain unclear. In this study, an HT-29 xenograft mouse model demonstrated that curcumol combined with oxaliplatin significantly suppressed tumor growth (Ki67↓) and microvessel density (CD31↓). In vitro assays revealed that curcumol dose dependently inhibited proliferation (MTT), migration (Transwell), and tube formation (CAM assay) in Caco-2/HT-29 and HUVEC cells. Mechanistically, curcumol downregulated OTUB1 expression, promoting TGFB1 degradation via the ubiquitin–proteasome pathway. OTUB1 overexpression activated the TGFB1/VEGF axis, enhancing cell invasiveness and angiogenesis—effects reversed by high-dose curcumol. These findings identify the OTUB1-TGFB1/VEGF axis as a key target of curcumol in inhibiting colon cancer angiogenesis, elucidating its anti-tumor mechanism and offering a novel therapeutic strategy for targeted treatment.

## 1. Introduction

The morbidity and mortality rates associated with colorectal cancer (CRC) are elevated across all subtypes [1,2]. Although surgical resection, chemotherapy, immunotherapy, and targeted therapy have significantly improved the 5-year survival rate in CRC patients, the risk of recurrence and metastasis persists. Therefore, gaining a comprehensive understanding of the molecular pathogenesis of colorectal cancer and identifying novel therapeutic targets is imperative.

One of the defining characteristics of all cancer types is angiogenesis, a process that facilitates the formation of new blood vessels to support tumor progression [3,4]. As a fundamental feature of cancer, angiogenesis plays a pivotal role in solid tumor development. Substantial evidence indicates that angiogenesis supplies the oxygen and nutrients essential for tumor cell survival, significantly contributing to cancer progression, particularly in colorectal cancer cell proliferation and metastasis. Anti-angiogenic therapy based on the concept of “starving tumors” has emerged as an attractive approach for treating diverse human malignancies, including colorectal cancer [5]. Several therapeutic agents targeting vascular endothelial growth factor (VEGF) have been developed, including bevacizumab, which was approved by the U.S. Food and Drug Administration (FDA) for metastatic colorectal cancer (mCRC) treatment [6]. These therapeutic agents inhibit VEGF from binding to its receptors (VEGFR-1 and VEGFR-2), thereby suppressing tumor angiogenesis, improving the tumor microenvironment, and enhancing chemotherapy drug delivery efficiency. Vascular normalization has emerged as a novel strategy in colorectal cancer treatment, involving the structural and functional remodeling of tumor blood vessels to resemble normal vasculature. This approach improves drug delivery efficiency while ensuring adequate oxygen supply [7]. Importantly, vascular normalization not only enhances treatment efficacy but also reduces drug resistance (DR) and metastatic potential. Angiogenesis in colorectal cancer represents a complex biological process involving multiple molecular pathways. Consequently, angiogenesis inhibitors provide dual benefits by simultaneously blocking angiogenesis and potentiating chemotherapy effects [8]. While anti-angiogenic therapy and vascular normalization strategies show considerable promise for colorectal cancer treatment, clinical applications of agents targeting NF-κB, VEGF, matrix metalloproteinases (MMPs), and HIF-1α may lead to adverse effects including hemorrhage, arterial thrombosis, and impaired wound healing [9]. These limitations have prompted growing interest in natural compounds with anti-angiogenic properties.

Traditional Chinese medicine has been actively utilized in anti-tumor therapy owing to its enhanced efficacy and reduced toxicity. The World Health Organization (WHO) has recognized the potential of various medicinal herbs and spices, recommending their incorporation into therapeutic regimens due to their favorable safety profiles [10].

Curcumol, a sesquiterpenoid active compound isolated from curcuma rhizome, demonstrates significant anti-tumor activity against various malignant epithelial tumors, including cervical, breast, and digestive tract cancers [11]. For instance, curcumol induces apoptosis in human gastric cancer AGS cells leading to cytotoxicity [12,13] and inhibiting growth and invasion of breast cancer [14]. The compound curcumol effectively overcomes docetaxel (DTX) resistance in prostate cancer by inhibiting the PI3K/Akt signaling pathway and suppressing the Warburg effect [15]. Curcumol down-regulates EBNA1 to exert anti-EVB-positive nasopharyngeal carcinoma invasion and metastasis inhibition [16]. The inhibitory effects of curcumol on the invasion, migration, and epithelial–mesenchymal transition of IGROV-1 and OVCAR-3 cells in ovarian cancer are mediated through the targeted inhibition of PAX8 [17]. Nevertheless, the potential anti-angiogenic and anti-metastatic effects of curcumol in colorectal cancer remain to be elucidated.

Ubiquitin modification significantly modulates vascular endothelial cell barrier function and inflammatory responses, consequently affecting blood vessel stability and permeability [18]. In the tumor microenvironment, ubiquitination modification plays a regulatory role in the formation and maturation of tumor blood vessels. Specifically, ubiquitination processes influence both the survival and differentiation of tumor vascular endothelial cells, as well as vascular stability and functionality [19]. These findings have led to growing interest in developing anti-angiogenic therapies that target ubiquitination pathways. OTUB1, a member of the OTU superfamily of deubiquitinating enzymes (DUBs), plays critical roles in diverse pathophysiological processes, including ferroptosis, carcinogenesis, DNA damage response, and immune regulation [20,21,22]. This enzyme functions by cleaving ubiquitin moieties from target proteins, thereby preventing their proteasomal degradation [23]. OTUB1 demonstrates elevated expression levels across multiple tumor types [24,25] and participates in cell cycle control and apoptotic regulation through protein degradation pathways. Furthermore, OTUB1 promotes hypoxia-induced glycolytic reprogramming and enhances DNA damage, ultimately influencing tumor progression and metastasis through multiple molecular mechanisms [26,27,28]. Notably, increased OTUB1 expression in colorectal cancer is associated with metastatic potential and unfavorable clinical outcomes. Mechanistically, OTUB1 facilitates cancer cell migration and invasion by regulating epithelial–mesenchymal transition (EMT) markers [29].

The protein TGFBI (transforming growth factor-beta induced) is associated with tumor development, and its stability is regulated by the processes of ubiquitination and deubiquitination [30,31]. Recent studies have identified a specific binding interaction between OTUB1 and TGFBI that is essential for OTUB1-mediated deubiquitination of TGFBI [32]. Our investigation demonstrates that through its OTU domain, OTUB1 interacts with multiple ubiquitin chains in the endoplasmic reticulum-associated degradation pathway, thereby impeding the degradation process of TGFBI. These findings provide new insights into the role of OTUB1 in tumor angiogenesis. Importantly, the OTUB1-TGFBI interaction may represent a potential therapeutic target for mediating the anti-angiogenic effects of curcumol.

This study investigated the anti-angiogenic effects of curcumol in colorectal cancer. In vivo experiments demonstrated that curcumol significantly inhibited tumor growth and angiogenesis in subcutaneous xenograft mouse models. Furthermore, we systematically evaluated the compound’s effects on proliferation, migration, invasion, and tube formation using human colorectal cancer cell lines (Caco-2 and HT-29) and human umbilical vein endothelial cells (HUVECs). Mechanistic studies revealed that curcumol modulates TGFBI ubiquitination by suppressing OTUB1-mediated protein stabilization, ultimately attenuating angiogenesis in colorectal cancer. Our findings elucidate the molecular mechanism by which curcumol exerts its anti-metastatic effects through the regulation of the OTUB1/TGFBI axis, highlighting its potential as a novel therapeutic agent for colorectal cancer treatment.

## 2. Results

### 2.1. Curcumol Inhibits Tumor Formation in Nude Mice with Subcutaneous Colon Cancer

**Figure 1:**
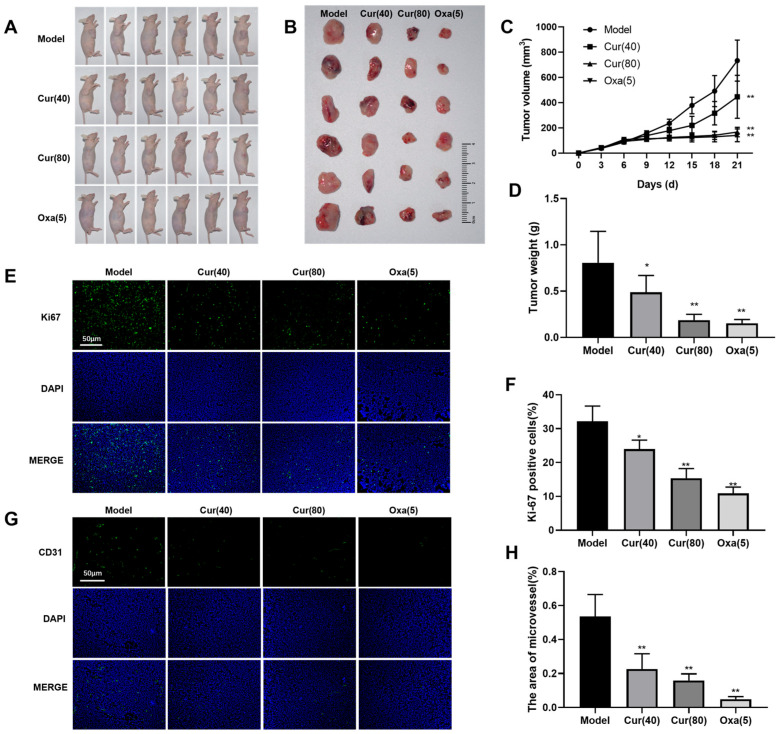
Curcumol inhibited the formation of subcutaneous tumors of HT-29 cells in nude mice. (**A**,**B**) Images of subcutaneous tumors in different groups of BALB/c nude mice. (Twenty-four tumor-bearing mice were randomly allocated into four groups: model control, low-dose curcumol (40 mg/kg), high-dose curcumol (80 mg/kg), and oxaliplatin positive control (5 mg/kg), with six mice per group). (**C**,**D**) Tumor growth curves, tumor weight and body weight of mice ((6 xenograft mice in each group)). (**E**,**F**) Quantitative immunofluorescence staining was used to assess the impact of curcumol on the proliferation marker Ki67 in xenograft tumor cells (scale bar = 50 μm, n = 3). (**G**,**H**) CD31 immunofluorescence staining was employed to evaluate microvascular density within implanted tumors across all groups(scale bar = 50 μm, n = 3). Data are presented as mean ± SD of three independent experiments. * *p* < 0.05 and ** *p* < 0.01, compared with the model group.

To assess the effects of curcumol on tumor growth in a colorectal cancer xenograft model, we initiated treatment on day 7 post-implantation. The model group received 90% propylene glycol via oral gavage, while the experimental groups were administered curcumol at different doses (40 mg/kg and 80 mg/kg) through the same route. The positive control group received 5 mg/kg oxaliplatin via tail vein injection. Following 14 days of treatment, tumors were excised, and their weights and volumes were measured (Figure 1A,B). Tumorigenesis assays revealed that all treatment groups showed significantly higher tumor inhibition rates in both volume and weight compared to the model group (* *p* < 0.05, ** *p* < 0.01; Figure 1C,D). We evaluated tumor cell proliferation using Ki67 immunofluorescence staining, which revealed distinct spatial heterogeneity in proliferative activity. While untreated tumors exhibited widespread Ki67-positive cells, all treatment groups showed significant reductions in the Ki67 proliferation index (PI), particularly in the high-dose curcumol and oxaliplatin groups (* *p* < 0.05, ** *p* < 0.01), demonstrating curcumol’s potent anti-proliferative effects (Figure 1E,F). Quantitative analysis of microvascular density via CD31 staining showed abundant CD31-positive vessels in untreated tumors, indicative of active angiogenesis. Both curcumol-treated and oxaliplatin control groups exhibited significantly fewer microvessels than the model group (** *p* < 0.01), suggesting that curcumol suppresses tumor angiogenesis, thereby limiting blood supply and inhibiting tumor growth (Figure 1G,H).

### 2.2. The Impact of Curcumol on the Colon Cancer Cell Lines Caco-2/HT-29 and Human Umbilical Vein Endothelial Cells (HUVECs) Was Investigated

**Figure 2:**
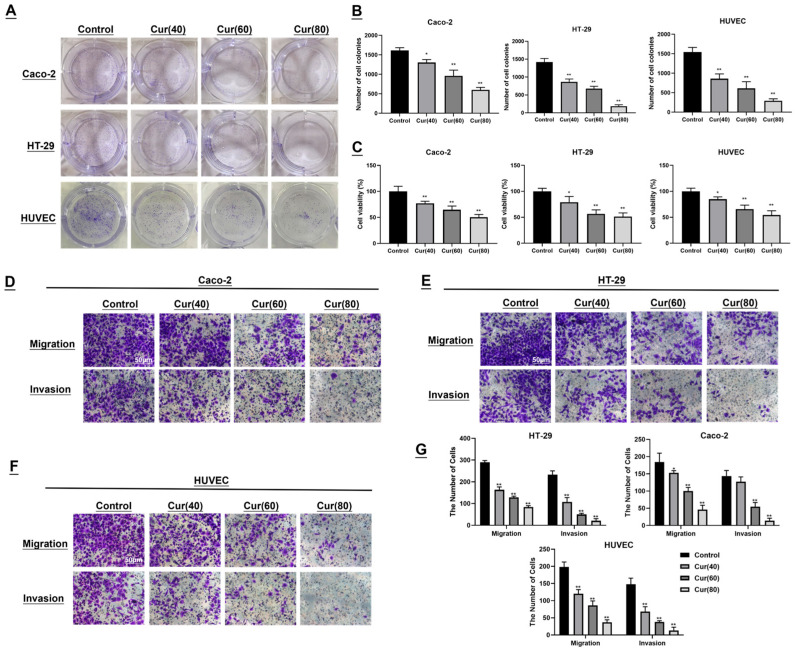
In vitro study of curcumol on colon cancer cells and human umbilical vein endothelial cells (HUVECs). (**A**,**B**) Curcumol-dependent effects of curcumol on colony formation in Caco-2, HT-29, and HUVEC cells. Colony formation rate = (number of colonies/number of seeded cells) × 100% (n = 3). (**C**) MTT assay demonstrated an inverse correlation between cell viability and curcumol concentration (n = 3). (**D**–**G**) Cell migration and invasion abilities were assessed using Transwell chambers. Crystal violet-stained cells represented migrated and invaded cells (scale bar = 50 μm, n = 3). Data are presented as mean ± SD of three independent experiments. * *p* < 0.05 and ** *p* < 0.01, compared with the control group.

To evaluate the biological effects of curcumol, colon cancer cell lines (HT-29 and Caco-2) and human umbilical vein endothelial cells (HUVECs) were exposed to increasing concentrations of curcumol (40, 60 and 80 μg/mL) for 24 h. Colony- formation assays revealed that curcumol treatment dose-dependently reduced both the size and number of Caco-2 and HT-29 colonies compared to untreated controls. Similarly, HUVEC viability decreased in a concentration-dependent manner following curcumol exposure (* *p* < 0.05, ** *p* < 0.01; Figure 2A,B). MTT assays further confirmed curcumol’s potent anti-proliferative effects on both colon cancer cells and HUVECs. Cell viability was significantly reduced in curcumol-treated groups compared to controls, with viability dropping below 50% at 80 μg/mL (* *p* < 0.05, ** *p* < 0.01), demonstrating a clear concentration-dependent response (Figure 2C). These results suggest that curcumol exhibits anti-angiogenic potential by suppressing endothelial cell proliferation. Transwell migration and invasion assays revealed that curcumol treatment markedly attenuated migratory and invasive capacities across all cell lines. Increasing curcumol concentrations correlated with progressively fewer transmigrated and invading cells (** *p* < 0.01; Figure 2D–G), indicating a dose-dependent suppression of metastatic potential in colorectal cancer.

### 2.3. Curcumol Reduces Angiogenesis in Subcutaneous HT-29 Cell Tumors in Nude Mice

**Figure 3:**
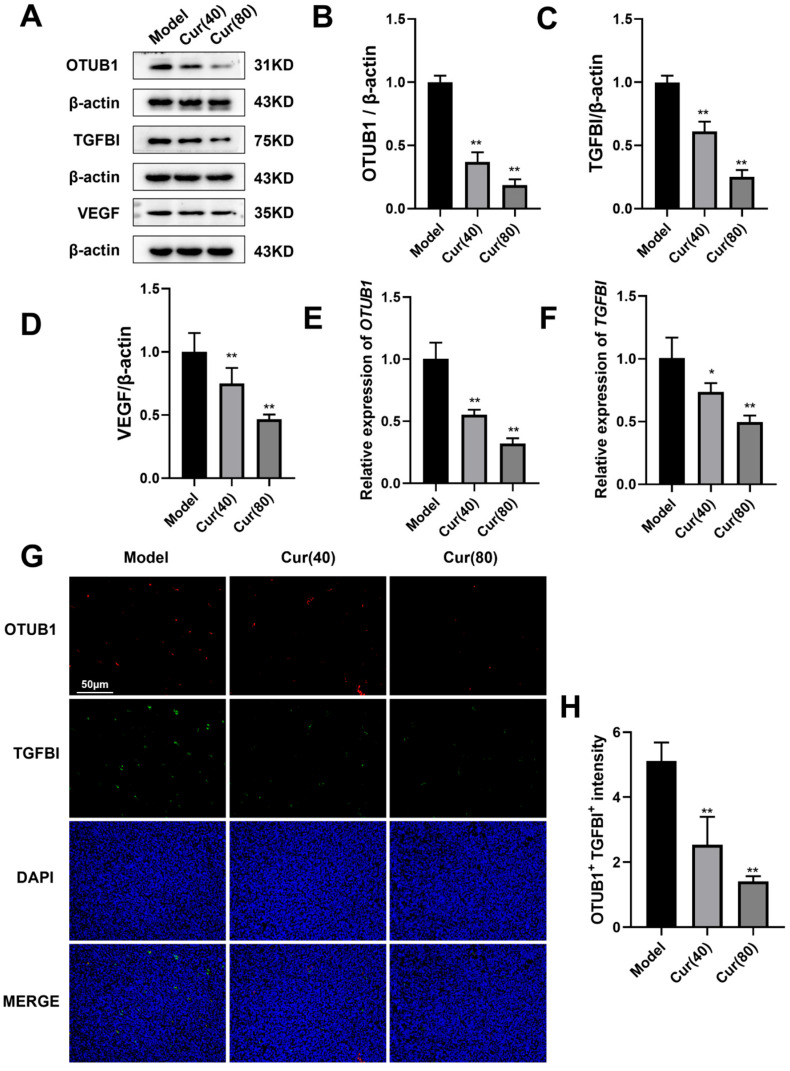
The expression levels of OTUB1, TGFBI, and VEGF in tumor tissues can be down-regulated by curcumol. (**A**–**D**) Western blot analysis of the effects of curcumol on the expression of OTUB1, TGFBI, and VEGF in xenograft tumor cells (n = 3). (**E**,**F**) Quantitative polymerase chain reaction (PCR) assessment of OTUB1 and TGFBI mRNA expression in tumor tissues (n = 3). (**G**,**H**) Immunofluorescence staining was utilized to examine the co-localization of OTUB1 and TGFB1 in the implanted tumor specimens of each group(scale bar = 50 μm, n = 3). The data are depicted as the arithmetic mean ± SD derived from at least three independent experiments. * *p* < 0.05 and ** *p* < 0.01 compared with the model group.

To elucidate the anti-angiogenic mechanism of curcumol in colorectal cancer, we analyzed angiogenesis-related protein expression in tumor tissues by Western blot. Untreated tumors (model group) exhibited an elevated expression of OTUB1, TGFBI, and VEGF (Figure 3A), suggesting their active involvement in tumor angiogenesis. In contrast, both curcumol-treated and oxaliplatin control groups showed significantly reduced expressions of these proteins (Figure 3B–D; ** *p* < 0.01). These findings indicate that curcumol effectively inhibits the expression of angiogenesis-related proteins and mitigates tumor angiogenesis and progression. qPCR analysis revealed that OTUB1 and TGFBI mRNA levels were substantially higher in untreated tumors compared to curcumol-treated samples (* *p* < 0.05, ** *p* < 0.01; Figure 3E,F), with high-dose curcumol showing the most pronounced suppression. This outcome implies that curcumol may exert its effects by downregulating OTUB1 and TGFBI gene expressions. To investigate the potential cooperation between OTUB1 and TGFBI in inhibiting tumor angiogenesis induced by curcumol, we employed immunofluorescence staining to assess the expression and co-localization of OTUB1 and TGFBI within the tumor tissues of nude mouse xenografts. Immunofluorescence staining demonstrated strong OTUB1-TGFBI co-localization in control tumors (Figure 3G), with OTUB1 distributed in nuclear/cytoplasmic compartments and TGFBI localized to the extracellular matrix and membrane regions. Curcumol treatment significantly attenuated both protein expression and its co-localization (** *p* < 0.01; Figure 3H), indicating that it disrupts OTUB1-TGFBI interactions that may be critical for tumor angiogenesis.

### 2.4. Curcumol Reduces the Angiogenesis of Caco-2/HT-29 Colorectal Cancer Cells

**Figure 4:**
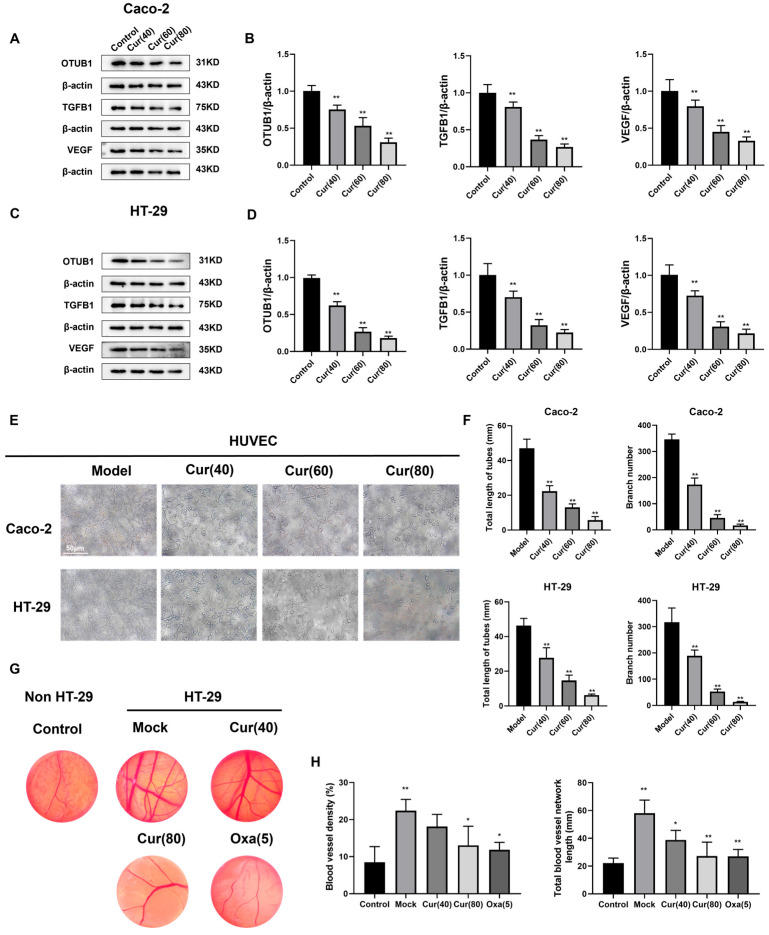
The expression levels of OTUB1, TGFBI, and VEGF were downregulated in Caco-2/HT-29 cells by curcumol, leading to the inhibition of angiogenesis in HUVEC cells. (**A**,**B**) The Western blot assay was employed to quantify the expression levels of OTUB1, TGFBI, and VEGF proteins in Caco-2 cells (n = 3, compared to the control group). (**C**,**D**) The Western blot assay was employed to quantify the expression levels of OTUB1, TGFBI, and VEGF proteins in HT-29 cells (n = 3, compared to the control group). (**E**,**F**) Following pharmacological treatment of tumor cells, the conditioned medium was collected to culture HUVEC cells. Subsequent tube formation assays were performed to quantify tubular network formation (scale bar = 50 μm, n = 3, compared to the model group). (**G**,**H**) After 48 h, chick chorioallantoic membranes (CAMs) were harvested to evaluate angiogenesis extent and vessel density (n = 3, compared to the control group). The data are depicted as the arithmetic mean ± SD derived from at least three independent experiments. * *p* < 0.05 and ** *p* < 0.01.

To further investigate the anti-angiogenic effects of curcumol in vitro, we collected conditioned media from Caco-2 and HT-29 cells treated with curcumol (40, 60, and 80 μg/mL) and co-cultured them with HUVECs for 24 h. Western blot analysis revealed that curcumol treatment significantly reduced the expression of angiogenesis-related proteins (OTUB1, TGFBI, and VEGF) in HUVECs compared to untreated controls (Figure 4A–D), demonstrating a concentration-dependent effect (** *p* < 0.01). These results were consistent with our in vivo observations. We evaluated curcumol’s anti-angiogenic potential using a tube formation assay. When plated on Matrigel, HUVECs formed characteristic tubular networks within 3–6 h. Quantitative analysis of tube length and branch points using image analysis software showed significant inhibition of tube formation in curcumol-treated groups (** *p* < 0.01; Figure 4E,F). The chick embryo develops an extensive network of allantoic membrane blood vessels during its growth, providing an ideal model for investigating angiogenesis. This model can be utilized to explore the mechanisms of angiogenesis and assess the angiogenic activity of pharmaceutical compounds. The chick embryo chorioallantoic membrane (CAM) assay was employed to further validate these findings. Both high-dose curcumol and oxaliplatin significantly reduced vessel density and branching in HT-29 xenografts compared to controls (* *p* < 0.05, ** *p* < 0.01; Figure 4G,H), confirming curcumol’s potent anti-angiogenic activity.

### 2.5. Curcumol Inhibits the Biological Activity of Human Colon Cancer Cell Lines Caco-2 and HT-29, Which Exhibit an Overexpression of OTUB1

**Figure 5:**
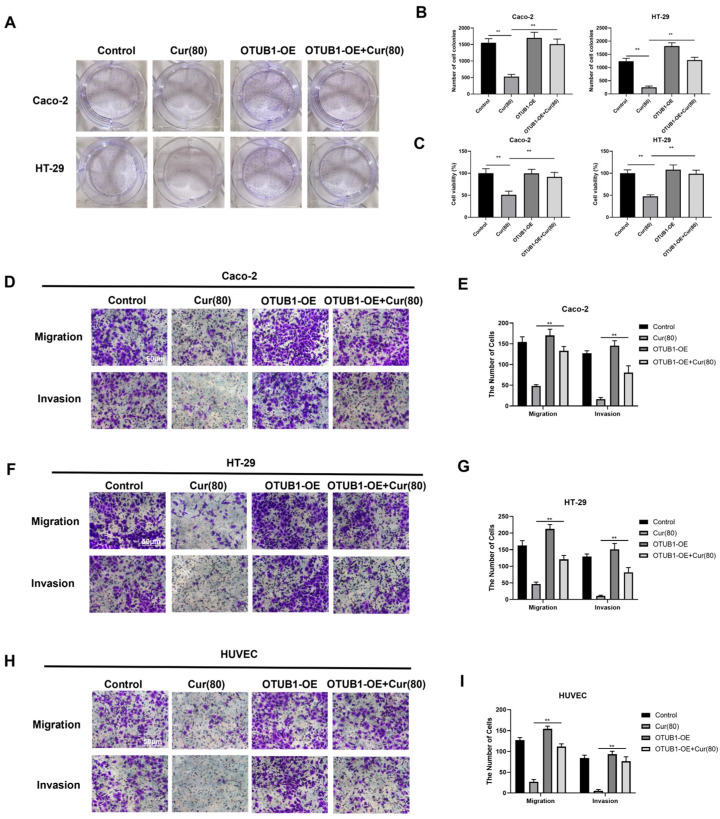
Curcumol is capable of suppressing the clonal formation and migratory potential of human colon cancer cells, Caco-2/HT-29, subsequent to the overexpression of OTUB1. (**A**,**B**) The clonogenic potential of Caco-2/HT-29 and HUVEC cells was assessed using a clonal formation assay (n = 3, ** *p* < 0.01, comparison between control group and high-dose curcumol treatment group; ** *p* < 0.01, comparison between Cur(80) group and OTUB1-OE + Cur(80) group). (**C**) MTT assay was utilized to evaluate cell viability (n = 3, comparison between control group and high-dose curcumol treatment group; comparison between Cur(80) group and OTUB1-OE + Cur(80) group). (**D**–**I**) Cell migration and invasion abilities were assessed using Transwell chambers (scale bar = 50 μm, n = 3, comparison between Cur(80) group and OTUB1-OE + Cur(80) group). Data are expressed as the mean ± SD of at least three independent experiments. ** *p* < 0.01.

To investigate curcumol’s mechanism of action through OTUB1 modulation in colorectal cancer and angiogenesis, we generated OTUB1 overexpression (OTUB1-OE) and control (Control-OE) plasmids, which were transfected into human colorectal cancer cell lines (HT-29 and Caco-2) and human umbilical vein endothelial cells (HUVECs). Cell proliferation and viability were assessed using MTT and clonogenic assays following drug treatment. The Control-OE group showed no significant difference in cell viability compared to empty vector controls. However, curcumol significantly inhibited proliferation in both HT-29 and Caco-2 cells (** *p* < 0.01). Notably, OTUB1-OE cells exhibited similar growth inhibition under curcumol treatment (** *p* < 0.01; Figure 5A–C), suggesting that curcumol mediates its anti-proliferative effects through OTUB1 downregulation. Transwell migration and invasion assays were performed to evaluate OTUB1’s role in cellular motility. While Control-OE transfection had no effect on migration or invasion in any cell type, OTUB1-OE cells showed significantly reduced migratory and invasive capacities following high-dose curcumol treatment (** *p* < 0.01; Figure 5D–G). These results indicate that curcumol potentiates its anti-metastatic effects by suppressing OTUB1 overexpression in colorectal cancer cells.

### 2.6. Curcumol Inhibits the Angiogenic Ability of Caco-2/HT-29 Colorectal Cancer Cells by Downregulating OTUB1 Expression

**Figure 6:**
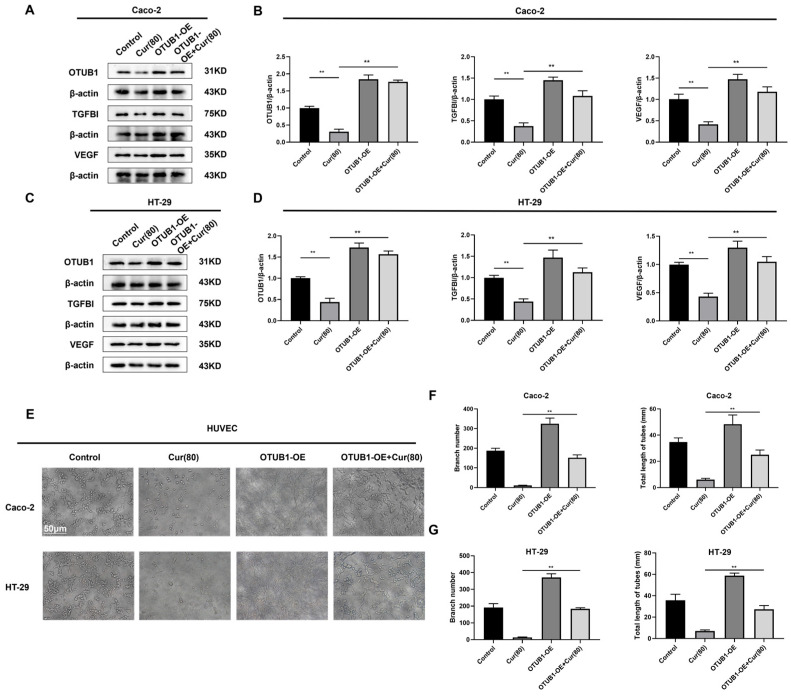
Curcumol can significantly downregulate the protein expression levels of OTUB1, TGFBI, and VEGF in Caco-2/HT-29 cells that overexpress OTUB1, thereby inhibiting the angiogenic potential of HUVEC cells. (**A**,**B**) The Western blot assay was employed to quantify the protein expression levels of OTUB1, TGFBI, and VEGF in Caco-2 cells that overexpress OTUB1 (n = 3, comparison between control group and high-dose curcumol treatment group; comparison between Cur(80) group and OTUB1-OE + Cur(80) group). (**C**,**D**) The Western blot assay was employed to quantify the protein expression levels of OTUB1, TGFBI, and VEGF in HT-29 cells that overexpress OTUB1 (n = 3, comparison between control group and Cur(80) group; comparison between Cur(80) group and OTUB1-OE + Cur(80) group). (**E**–**G**) The HUVEC cells were treated with the supernatant obtained after drug treatment. The tubule formation assay of the number of formed tubules (scale bar = 50 μm, n = 3, comparison between the Cur(80) group and the OTUB1-OE + Cur(80) group). Data are expressed as the mean ± SD of at least three independent experiments. ** *p* < 0.01.

To elucidate the role of OTUB1 in colon cancer cells and angiogenesis, we constructed the OTUB1-OE and Control-OE plasmids as previously described, followed by transfection into human colon cancer cell lines Caco-2 and HT-29. Western blot analysis demonstrated that curcumol treatment significantly reduced expression of angiogenesis-related proteins (OTUB1, TGFBI, and VEGF) in Control-OE cells, confirming the empty vector’s neutral effect on curcumol’s anti-angiogenic activity. Notably, OTUB1-OE cells exhibited markedly elevated levels of these pro-angiogenic proteins compared to controls (** *p* < 0.01). However, curcumol treatment effectively counteracted this upregulation (** *p* < 0.01; Figure 6A–D), demonstrating its capacity to suppress OTUB1-mediated angiogenic signaling. These findings indicate that while the overexpression of OTUB1 promotes angiogenesis-related protein expression, curcumol serves as an effective inhibitor—an assertion supported by subcutaneous tumor experiments conducted in nude mice. Tube formation assays revealed that OTUB1 overexpression significantly enhanced HUVEC angiogenic capacity, as evidenced by increased tube length, total network length, and branching points (** *p* < 0.01; Figure 6E–G). Curcumol treatment reversed these pro-angiogenic effects, confirming that its anti-angiogenic activity operates through OTUB1 suppression.

### 2.7. Curcumol Regulates the Deubiquitination of TGFBI by OTUB1 to Inhibit Angiogenesis Induced by Colorectal Cancer

**Figure 7:**
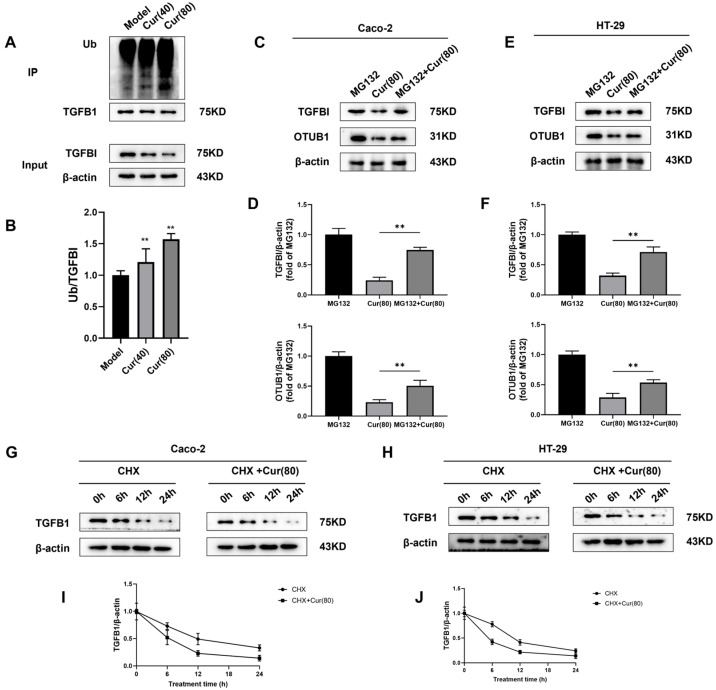
The presence of curcumol inhibits the deubiquitination activity of OTUB1 on TGFBI, thereby enhancing the ubiquitination-mediated degradation process of TGFBI. (**A**,**B**) Immunoprecipitation (IP) analysis of the interaction of TGFBI (n = 3, ** *p* < 0.01,compared to the model group). (**C**,**D**) After treatment with MG132, the impact of curcumol on the expression level of OTUB1 in Caco-2 cells and its effect on TGFBI ubiquitination were assessed through Western blot analysis (n = 3, comparison between Cur(80) group and MG132 + Cur(80) group). (**E**,**F**) After treatment with MG132, the impact of curcumol on the expression level of OTUB1 in HT-29 cells and its effect on TGFBI ubiquitination were assessed through Western blot analysis (n = 3, comparison between Cur(80) group and MG132 + Cur(80) group). (**G**–**J**) Western blot analysis of the half-life of TGFBI protein in Caco-2 and HT-29 cells after curcumol treatment (n = 3). Data are expressed as the mean ± SD of at least three independent experiments. ** *p* < 0.01.

In order to investigate the curcumol mechanism in inhibiting colon cancer angiogenesis through OTUB1 downregulation and TGFBI ubiquitination, we performed immunoprecipitation (IP) and Western blot analyses. Ubiquitinated TGFBI appeared as higher molecular weight bands, with curcumol treatment significantly increasing the Ub/TGFBI ratio compared to controls (** *p* < 0.01, Figure 7A,B), indicating enhanced TGFBI ubiquitination and subsequent degradation. We systematically investigated OTUB1’s role in TGFBI stabilization using Caco-2/HT-29 cells. As an OTU deubiquitinase family member, OTUB1 typically stabilizes substrate proteins. Western blot analysis revealed that curcumol treatment significantly reduced TGFBI ubiquitination levels compared to the proteasome inhibitor MG132 control (** *p* < 0.01; Figure 7C–F). This effect was reversed by MG132 pretreatment, demonstrating that curcumol promotes TGFBI degradation via the ubiquitin–proteasome pathway by inhibiting OTUB1-mediated deubiquitination. Protein stability assays using cycloheximide (CHX) chase experiments showed that curcumol treatment significantly shortened TGFBI’s half-life in both Caco-2 and HT-29 cells (Figure 7G–J), confirming accelerated protein degradation. These results establish that curcumol inhibits tumor angiogenesis by disrupting the OTUB1-TGFBI axis through enhanced ubiquitination and the degradation of TGFBI.

## 3. Discussion

Nearly 90% of malignant tumors succumb to recurrence and metastasis, as tumor angiogenesis plays a crucial role in the growth and spread of tumors. Tumor cells release various pro-angiogenic factors, such as vascular endothelial growth factor (VEGF) and stromal cell-derived factor (SDF-1), which stimulate the proliferation and movement of endothelial cells in nearby normal tissues [5]. This angiogenic cascade facilitates neovascularization to support tumor oxygenation and nutrient supply while simultaneously establishing metastatic routes. Given this pathophysiology, anti-angiogenic therapy has emerged as a cornerstone of oncologic treatment. While clinical agents, like bevacizumab, demonstrate efficacy, their utility is limited by adverse effects (thrombosis, hemorrhage) and acquired resistance [33]. Consequently, the development of novel anti-angiogenic agents with improved safety profiles remains an urgent priority. Natural products derived from medicinal plants, fungi, and microbial sources show particular promise as reservoirs of potent yet low-toxicity anti-angiogenic compounds [34].

Our research group is dedicated to investigating the impact of traditional Chinese medicines on tumor angiogenesis, particularly in promoting blood circulation and resolving blood stasis. Curcumol, a bioactive component of curcuma and a representative Chinese medicinal compound, has demonstrated anticancer activity against various malignancies, including gastric cancer [12], breast cancer [14], and nasopharyngeal carcinoma [16]. This study elucidates the mechanism by which curcumol inhibits angiogenesis and metastasis in colon cancer via the OTUB1/TGFB1 pathway. We report, for the first time, that curcumol suppresses colorectal cancer progression by downregulating OTUB1 expression, thereby targeting tumor-associated angiogenesis. Furthermore, curcumol exhibits potential as a novel, low-toxicity anti-angiogenic agent. Our findings indicate that curcumol significantly inhibits the migration and invasion of colon cancer cells (HT-29 and Caco-2) and disrupts tubule formation, migration, and invasion in HUVECs. Additionally, curcumol impedes subcutaneous tumor growth in nude mice. Immunohistochemical analysis using CD31 and Ki67 markers revealed that curcumol reduces tumor proliferation and microvessel density in a dose-dependent manner, with high-dose curcumol demonstrating efficacy comparable to oxaliplatin. Gupta et al. [35] reported that cancer-induced angiogenesis is primarily driven by pro-angiogenic factors secreted by cancer cells, such as VEGF [36,37], which stimulate endothelial cell activation, thereby facilitating tumor invasion and metastasis [38,39]. To further investigate this mechanism, we assessed angiogenesis-related proteins and conducted inhibition assays. Our results confirmed that curcumol effectively suppresses colon cancer cell (HT-29 and Caco-2) and HUVEC migration, invasion, and tubule formation. Mechanistic studies revealed that curcumol downregulates OTUB1, TGFB1, and VEGF expression in both colon cancer and vascular endothelial cells. These in vitro findings were corroborated by in vivo experiments in a nude mouse xenograft model.

Studies have demonstrated that elevated OTUB1 expression in colorectal cancer patients correlates with poor prognosis, increased tumor aggressiveness, and higher recurrence rates [28]. Therefore, OTUB1 represents a potential biomarker for evaluating colorectal cancer progression and predicting patient outcomes.Theinhibition of OTUB1 activity or the downregulation of its expression may contribute to controlling colorectal cancer progression. As a key member of the OTU deubiquitinase subfamily, OTUB1 regulates multiple critical pathways involved in carcinogenesis [40]. Increased OTUB1 expression in the tumor microenvironment influences cytokine secretion and matrix metalloproteinase (MMP) release, modifies the tumor microenvironment structure, and enhances endothelial cell migration and proliferation, thereby promoting angiogenesis [41,42,43]. Through its pro-angiogenic effects, OTUB1 may further increase tumor invasiveness. Tumor neovascularization provides a route for cancer cell dissemination, facilitating distant metastasis. Our study confirmed that OTUB1 overexpression upregulates TGFB1 and VEGF expression, enhancing the proliferative, migratory, and invasive capacities of human colon cancer cells (HT-29 and Caco-2), as well as the tubule formation ability of HUVECs. Curcumol treatment counteracted these effects by suppressing TGFB1 and VEGF expression following OTUB1 overexpression, reducing colon cancer cell proliferation, migration, and invasion and inhibiting HUVEC tubule formation. These findings demonstrate that curcumol suppresses colorectal cancer proliferation and angiogenesis by targeting OTUB1 expression.

**Figure 8:**
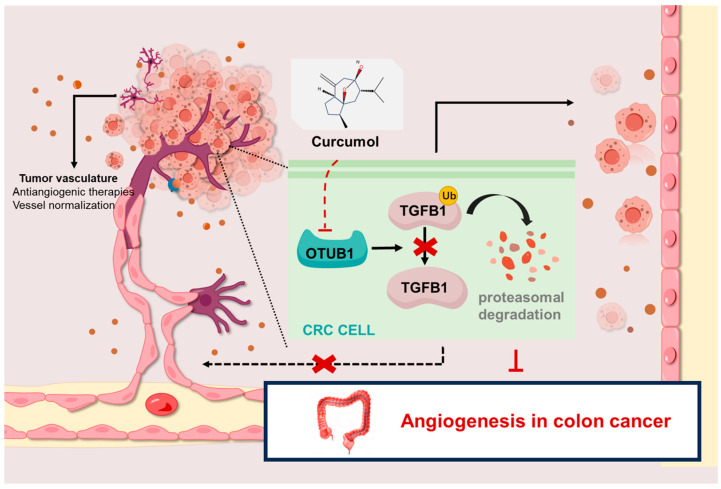
Model showing that curcumol inhibits the OTUB1 (OTUdomain, ubiquitin aldehyde binding 1)-mediated deubiquitination of TGFBI (transforming growth factor-beta induced), promoting its ubiquitination degradation and thereby suppressing colon cancer angiogenesis.

TGFBI (transforming growth factor- beta induced), an extracellular matrix protein originally identified as a responsive gene to the TGF-β signaling pathway, plays crucial roles in regulating cell adhesion, migration, and proliferation [44,45,46]. Accumulating evidence demonstrates that TGFBI displays dysregulated expression patterns across multiple malignancies, with its expression levels exhibiting strong correlations with tumor invasiveness and metastatic potential. Notably, TGFBI exhibits context-dependent oncogenic functions that vary among different cancer types. For instance, in colorectal cancer, TGFBI overexpression is associated with high-grade tumors, enhancing cancer cell invasiveness and altering metastatic characteristics while paradoxically suppressing liver metastasis. In gastric cancer, elevated TGFBI expression significantly correlates with advanced clinical stages, high metastatic potential, and poor prognosis [47,48]. At the molecular level, TGFBI promotes tumor progression through multiple mechanisms. It synergizes with pro-angiogenic factors, such as VEGF, to enhance tumor angiogenesis. Through the integrin-mediated activation of FAK and PI3K/Akt signaling pathways, TGFBI facilitates vascular endothelial cell migration. Additionally, it modulates matrix metalloproteinase (MMP) activity to remodel the extracellular matrix, thereby promoting neovascularization [49]. Recent studies further revealed that TGFBI may indirectly regulate cell migration and invasion by modulating the expression or activity of the deubiquitinating enzyme OTUB1 [32]. Functional studies have demonstrated that TGFBI silencing significantly inhibits endothelial cell proliferation and vascular sprouting, whereas exogenous TGFBI treatment can reverse these effects, underscoring its pivotal role in tumor angiogenesis [50]. This study demonstrates that OTUB1 not only maintains TGFBI stability during angiogenesis but also promotes the overexpression of pro-angiogenic factors including VEGF. Our data reveal that TGFBI expression is positively regulated by OTUB1. Immunoprecipitation (IP) combined with Western blot analysis confirmed that curcumol disrupts the interaction between these two proteins. Mechanistic studies that suggest this regulation occurs through ubiquitination/deubiquitination homeostasis. Using ubiquitin-specific monoclonal antibodies under proteasome inhibition (MG132 treatment), we observed that curcumol significantly enhances TGFBI ubiquitination and subsequent degradation by suppressing OTUB1’s deubiquitinating activity. Kinetic analysis further revealed that curcumol treatment markedly reduces the half-life of TGFBI protein in both Caco-2 and HT-29 cell lines, indicating that the compound likely exerts its effects by promoting TGFBI ubiquitination while counteracting OTUB1-mediated deubiquitination protection. A detailed schematic of the mechanism is presented in Figure 8.These findings establish that: TGFBI stability depends on OTUB1-mediated deubiquitination protection. Curcumol compromises this protective mechanism, rendering TGFBI more susceptible to ubiquitin–proteasomal degradation. The OTUB1-TGFBI regulatory axis plays a crucial role in angiogenesis. This work not only elucidates a novel molecular mechanism in angiogenesis regulation but also identifies OTUB1 as a promising therapeutic target for developing protein stability-based interventions in angiogenesis-related disorders.

In conclusion, our study provides the first elucidation of the molecular mechanism by which curcumol suppresses colorectal cancer angiogenesis through downregulating OTUB1 expression, thereby blocking its deubiquitinating effect on TGFBI and promoting TGFBI ubiquitination-mediated degradation. These findings offer experimental evidence supporting curcumol as a potential targeted therapeutic agent against metastatic colorectal cancer. However, the current research has several limitations: (1) insufficient mechanistic validation (e.g., lack of loss-of-function experiments via OTUB1 knockout); (2) undefined site specificity (unidentified critical ubiquitination sites mediating OTUB1-TGFBI interaction); and (3) unverified direct binding between curcumol and OTUB1 or its enzymatic activity modulation, which requires further validation through binding assays (e.g., surface plasmon resonance [SPR]) or enzyme activity tests. Future studies should employ structural biology analyses of OTUB1-TGFBI protein interaction, biochemical validation of direct curcumol–OTUB1 binding, and dose-dependent evaluation of angiogenesis inhibition in both in vitro and in vivo models to refine the mechanistic understanding.

## 4. Materials and Methods

### 4.1. Chemical Substances and Reagents

The purified curcumol (>99%) (HY-N2259) was purchased from MCE (Monmouth Junction, NJ, USA) and dissolved in 0.1% dimethyl sulfoxide (DMSO). Ub, anti-OTUB1, and anti-rabbit IgG were sourced from Cell Signaling Technology (CST) in London, UK. The anti-TGFBI and anti-beta-actin antibodies were obtained from Affinity Biosciences located in Changzhou, Jiangsu, China. Oxaliplatin was obtained from Sigma (O9512, Shanghai, China). Fetal bovine serum, Rosewell Park Memorial Institute (RPMI)-1640 culture medium, trypsin digestive fluid, and penicillin/streptomycin combination preparations were acquired from Gibco (Grand Island, NY, USA). Transwell BD-Matrigel Basement Membrane Matrix Inserts (BD Biosciences, San Jose, CA, USA), Bovine Collagen Type II (Chondrex, Redmond, WA, USA), penicillin/streptomycin 100 units solution, dimethyl sulfoxide (DMSO), HEPES buffer solution, propidium iodide (Sigma-Aldrich, Gillingham, UK), calcium chloride, actinomycin D, sodium bicarbonate, sodium chloride, and disodium hydrogen phosphate were purchased from Merck & Co., Kenilworth, NJ, USA. Reagents utilized in this study comprised MG132 (Selleck, Shanghai, China) and cycloheximide (CHX) (Selleck, Shanghai, China). MG132 and CHX were dissolved in dimethylsulfoxide (Solarbio, Beijing, China).

### 4.2. Cell Line Cultivation and OTUB1 Overexpression

The colon cancer cell lines (Caco-2 and HT-29 cells) and human umbilical vein endothelial cells (HUVECs) were obtained from the Cell Resource Center (ATCC) of the Chinese Academy of Sciences. Caco-2 cells were cultured in Dulbecco’s Modified Eagle Medium (DMEM) supplemented with 20% fetal bovine serum (FBS) and 1% penicillin/streptomycin (Thermo Scientific Hyclone, Logan, UT, USA). HUVEC cells were cultured in Dulbecco’s Modified Eagle Medium/Nutrient Mixture F-12 Ham (DEME/F12) containing 1% endothelial cell growth supplement (ECGS, Sigma E2759), 10% FBS (Sigma-Aldrich), and 1% antibiotic solution (penicillin/streptomycin). The cell incubator was maintained at a humidified atmosphere of 37 °C with a CO_2_ concentration of 5%. The OTUB1 overexpression plasmid and control plasmid were provided by Cusabio, using Lipofectamine 2000 for a duration of 48 h.

### 4.3. Animal Model and Categorization

The BALB/c-nu male nude mice used in this study were approximately 5 weeks old and weighed around 20 g. They were obtained from Qinglongshan Animal Husbandry Farm, Jiangning District, Nanjing, China (animal license number SYXK (SU) 2018-0049). The mice were housed in the Laboratory Animal Center at Nanjing University of Chinese Medicine under controlled conditions with a room temperature of 18–22 °C, relative humidity of 50–60%, and a 12-h light–dark cycle. They had ad libitum access to food and water and were kept in an independent SPF ventilation system with barrier protection. All animal experiments followed the guidelines set by the Ethics Committee of the Experimental Animal Center at Nanjing University of Chinese Medicine (Experimental Animal Ethics A220607).The HT-29 subcutaneous tumor model was established by injecting a suspension containing HT-29 cells at a concentration of 1 × 10^7^/mL into the subcutaneous tissue on the side of the shoulder blade in each nude mouse. Tumor volume was measured every two days after model establishment until tumors reached or exceeded a diameter of 0.5 cm (starting from day 6), indicating successful tumor growth. A total of twenty-four mice with successfully established tumor models were sorted based on their weight from lightest to heaviest and randomly divided into four groups, each consisting of six mice: model group (0.2 mL; vehicle control—-propylene glycol solution), low-dose curcumol group (40 mg/kg), high-dose curcumol group (80 mg/kg) [51], and positive drug group (oxaliplatin, injected intravenously at a dose of 5 mg/kg once weekly for fifteen days).

### 4.4. Immunofluorescence Staining of Ki67

The subcutaneous implant tumor tissue samples were fixed post-resection, rinsed with phosphate-buffered saline (PBS), subjected to high-temperature antigen retrieval, and subsequently sealed using a solution of fetal bovine serum albumin. The samples were then incubated with anti-Ki67 antibody (ab15580) at room temperature for 1–2 h. After washing with PBS, fluorescently labeled secondary antibodies were added and incubated for 1 h. DAPI staining was performed to visualize the nuclei before sealing the slides following the washing steps. Fluorescence microscopy was employed to observe and record the results, which were further quantitatively analyzed using ImageJ software (1.8.0_322).

### 4.5. Immunofluorescent Analysis of CD31

The excised tissue samples were fixed and embedded, followed by sectioning with 4% formaldehyde (PFA) and 1% saponin in phosphate-buffered saline (PBS). Overnight closure was performed using PBS containing 1% fetal bovine serum albumin (BSA) and 10% goat serum. The Cd31-specific antibody (recombinant anti-CD31 antibody [EPR17259] (ab182981)) was added and incubated overnight, while the corresponding fluorescently labeled secondary antibody (anti-rabbit IgG) was utilized. DAPI staining was employed for nuclear visualization, and the images were observed under a fluorescence microscope before being quantitatively analyzed using ImageJ software.

### 4.6. Experiment on Colony Formation

A single-cell suspension was prepared by combining developing colon cancer cells (HT-29 and Caco-2 cells) with human umbilical vein endothelial cells (HUVECs). The 6-well plates were incubated in a cell incubator at 37 °C and 5% CO_2_ for 24 h. Following cell adhesion, gradient concentrations of curcumol (40, 60, 80 μg/mL) were added, and the medium containing different concentrations of curcumol was changed every 3 days. At experimental endpoints, cells were fixed with 4% paraformaldehyde (15–30 min), stained with 0.5% crystal violet (20 min), washed with PBS, and air-dried before microscopic enumeration of colonies. Colony formation efficiency was calculated as (number of colonies/number of seeded cells) × 100%. Each experiment was repeated three times.

### 4.7. Methyl Thiazolyl Tetrazolium (MTT) Assay for Cell Viability

A single-cell suspension was prepared by combining developing colon cancer cells (HT-29 and Caco-2 cells) with human umbilical vein endothelial cells (HUVECs). The cell suspension was adjusted to the appropriate concentration and inoculated into a 96-well plate. Subsequently, the cells were treated with varying doses of curcumol. After a specific incubation period, MTT solution and DMSO solution were sequentially added, followed by determination of the optical density (OD) value for each well using an enzymometer to assess cellular activity.

### 4.8. Transwell Assay for Cellular Migration and Invasion

HT-29, Caco-2, and HUVECs were cultured in 200 μL medium with varying curcumol concentrations (40, 60, 80 μg/mL) in Transwell chambers. Lower chamber cells were kept in 600 μL medium with 10% FBS. Different concentrations of curcumin were administered to three wells. For invasion assays, Matrigel-coated surfaces mimicked the extracellular matrix. Post-incubation, cells were fixed in methanol and stained with 0.1% crystal violet and were then counted under an inverted microscope. Each experiment was conducted in triplicate.

### 4.9. The Immunofluorescence of Tumor Tissue

The excised subcutaneous implant tumor tissue samples were fixed at room temperature for 30 min, washed with PBS, subjected to antigen retrieval at high temperature, and subsequently blocked with fetal bovine serum albumin solution. Recombinant anti-TGFBI antibody [EPR12079(B)] (ab169771) and anti-OTUB1 antibody (ab198214) were added to the samples and incubated at room temperature for 1–2 h. After washing with PBS, fluorescently labeled secondary antibodies were applied and incubated for 1 h. The nuclei were stained with DAPI, followed by sealing of the slides after washing. Fluorescence microscopy was employed to observe and document the results, which were then co-localized and analyzed using ImageJ software.

### 4.10. Tubule Formation Assay

The HUVEC cells in optimal condition were carefully chosen, and the diluted Matrigel matrix adhesive was added to each well of the 48-well plate, which was then incubated at 37° C for 1 h. Subsequently, each well was inoculated with a density of 1 × 10^5^ HUVEC cells. Following a culture period of 12 h, the tubes were captured using an inverted microscope, and three random fields of view were selected for counting tubule branch points.

### 4.11. Chick Embryo Allantoic Membrane (CAM) Experiment

Chicken embryos aged 4 to 7 days post-fertilization were carefully selected for incubation. Small openings were created on the opposite side of the egg’s air chamber to expose the allantoic membrane, followed by inoculation of the prepared cell suspension into the allantoic membrane. The eggs were then returned to the incubator and maintained at a constant temperature (37 °C) and humidity (60%). Subsequently, drugs were injected into the allantoic membrane, and regular observations were conducted during culture. After 48 h, neovascularization was quantified by measuring parameters such as blood vessel density, total vascular network length, branch number, and total vascular segment in different groups: no HT-29 cell group, HT-29 cell group, HT-29 cell + low-concentration drug group, HT-29 cell + medium-concentration drug group, and HT-29 cell + high-concentration drug group.

### 4.12. Western Blot

Cells and tumor tissues from each group were harvested and lysed with a RIPA buffer to extract total protein. Protein concentration was quantified using the BCA assay. After denaturation, 10 μg of protein per sample was separated by electrophoresis and transferred to a PVDF membrane (1 h). The membrane was blocked with 5% BSA and incubated overnight at 4 °C with a primary antibody (1:1000 dilution), followed by a 2-h incubation with an HRP-conjugated secondary antibody (1:5000 dilution). β-actin was used as the loading control. Protein bands were visualized, and band intensities were quantified using ImageJ software. Target protein expression levels were normalized to β-actin.

### 4.13. Quantitative qRT-PCR

The RNA was extracted using the Trizol reagent, and cDNA synthesis was performed using the Prime Script First Strand cDNA Synthesis Kit. Following the manufacturer’s instructions, qRT-PCR was conducted utilizing SYBR Green technology on a Light Cycler 1.5 system, and data analysis was carried out employing the 2^−ΔΔCt^ method. The primer sequences applied in the qPCR experiments are shown in Table 1.

### 4.14. Immunoprecipitation (IP)

The human colon cancer cells were collected and lysed using cell lysate under non-denaturing conditions to preserve protein–protein interactions. After cell lysis, the RIPA lysis buffer containing protease inhibitor is used for dissolution. Then, a TGFB1-specific antibody is added and incubated overnight at 4 °C. Protein A/G agarose beads are subsequently added and incubated for 2 h at 4 °C. The samples are washed three times with a PBS buffer containing 0.1% Triton X-100, followed by protein elution using a sample buffer and SDS-PAGE for Western blot analysis. After capturing the image using ImageJ software, the gray value of each band was quantified. ImageJ software was employed for analyzing the gray values of each band, while β-actin served as an internal reference for calculating the expression level of the target protein.

### 4.15. Determination of the Half-Life of TGFBI Protein

In the TGFBI protein half-life assay, Caco-2 and HT-29 cells were treated with CHX (30 μm/mL) and a high dose of curcumol, respectively, for the designated duration. Subsequently, samples were collected and subjected to Western blot analysis to determine the expression level of TGFBI.

### 4.16. Ubiquitination Experiments in Cells

Both Caco-2 and HT-29 cells were treated with MG132 for a duration of 6 h. Cells were harvested and lysed in an ice-cold lysis buffer for 30 min on ice. After centrifugation at 14,000× *g* for 15 min at 4 °C, the supernatant was collected as the total cell lysate. For immunoprecipitation, 500 μg of total protein was incubated with 2 μg of the specific primary antibody against the target protein overnight at 4 °C with gentle rotation. Then, 20 μL of protein A/G agarose beads was added and incubated for an additional 2 h at 4 °C. The immunocomplexes were collected by centrifugation at 1000× *g* for 5 min and washed five times with a lysis buffer. The precipitates were eluted in a 2× SDS sample buffer by boiling for 5 min. The immunoprecipitated proteins were then separated by SDS-PAGE and transferred to a PVDF or nitrocellulose membrane for Western blot analysis.

### 4.17. Statistical Analysis

The data analysis in this study was performed using GraphPad Prism 7.0 software, with each experiment being repeated three times. One-way ANOVA and Dunnett’s multiple comparison test were employed to assess the differences between groups, and the results were reported as mean ± standard deviation (SD). Statistical significance was defined as * *p* < 0.05 and ** *p* < 0.01.

## 5. Conclusions

Based on current research evidence, this study elucidates the molecular mechanism by which curcumol suppresses angiogenesis and metastasis in colorectal cancer through modulation of the OTUB1/TGFBI signaling pathway. Specifically, curcumol significantly downregulates OTUB1 expression, thereby blocking its deubiquitinating effect on TGFBI and promoting TGFBI ubiquitination-mediated degradation. This mechanism ultimately leads to marked inhibition of angiogenesis, migration, invasion, and metastatic potential in colorectal cancer cells. These findings not only provide novel molecular insights into the anti-tumor effects of curcumol but also establish a scientific foundation for its clinical translation.

From a translational medicine perspective, curcumol demonstrates remarkable antitumor activity and promising development potential. To facilitate its clinical application, subsequent research should focus on the following priorities: (1) structural optimization of curcumol through molecular docking techniques and computer-aided drug design to enhance its target specificity and bioavailability; (2) systematic preclinical pharmacodynamic evaluations, including pharmacokinetic profiling and in vitro/in vivo antitumor efficacy assessments, complemented by comprehensive toxicological studies; and (3) well-designed phase I/II clinical trials with particular emphasis on evaluating safety profiles and preliminary therapeutic efficacy in patients with advanced colorectal cancer. The advancement of these research directions will accelerate the translational process of curcumol from bench to bedside, potentially providing a novel targeted therapeutic option for colorectal cancer patients.

## Figures and Tables

**Table 1:** PCR primer sequences.

Gene Name	Primer Sequences (5′–3′)
TGFBI-F	TCAAAGATGGTGTCCCTCGC
TGFBI-R	ACATCCGGTCCATGGTGAAC
OTUB1-F	GCTGTGCAGAATCCTCTGGT
OTUB1-R	AAGCCAAACGCTCGGTAGAA

## Data Availability

Data will be made available upon request.

## Abbreviations

CAM	chick embryo allantoic membrane
CHX	cycloheximide
DMEM	Dulbecco’s Modified Eagle Medium
DMSO	dimethyl sulfoxide
DR	Drug Resistance
DTX	docetaxel
DUB	deubiquitinating
EMT	epithelial-mesenchymal transition
FBS	fetal bovine serum
PFA	formaldehyde
HUVEC	human umbilical vein endothelial cells
mCRC	metastatic colorectal cancer
MMP	matrix metalloproteinase
MTT	Methyl thiazolyl tetrazolium
PBS	phosphate-buffered saline
SD	standard deviation
SDF-1	stromal cell-derived factor
WHO	the World Health Organization
VEGF	vascular endothelial growth factor
